# GalaxyRefine2: simultaneous refinement of inaccurate local regions and overall protein structure

**DOI:** 10.1093/nar/gkz288

**Published:** 2019-04-19

**Authors:** Gyu Rie Lee, Jonghun Won, Lim Heo, Chaok Seok

**Affiliations:** Department of Chemistry, Seoul National University, Seoul 08826, Korea

## Abstract

The 3D structure of a protein can be predicted from its amino acid sequence with high accuracy for a large fraction of cases because of the availability of large quantities of experimental data and the advance of computational algorithms. Recently, deep learning methods exploiting the coevolution information obtained by comparing related protein sequences have been successfully used to generate highly accurate model structures even in the absence of template structure information. However, structures predicted based on either template structures or related sequences require further improvement in regions for which information is missing. Refining a predicted protein structure with insufficient information on certain regions is critical because these regions may be connected to functional specificity that is not conserved among related proteins. The GalaxyRefine2 web server, freely available via http://galaxy.seoklab.org/refine2, is an upgraded version of the GalaxyRefine protein structure refinement server and reflects recent developments successfully tested through CASP blind prediction experiments. This method adopts an iterative optimization approach involving various structure move sets to refine both local and global structures. The estimation of local error and hybridization of available homolog structures are also employed for effective conformation search.

## INTRODUCTION

Template-based protein structure prediction methods can generate accurate protein models when sufficiently similar structural templates are available (1). Recently, template-free methods have also been able to produce highly accurate models (2,3) because of advances in coevolution analysis (4) and deep learning algorithms (5,6), which can extract protein structure information from protein sequences. However, the predicted protein structures may be inaccurate in regions for which there is not sufficient experimental data regarding template structures or related sequences available. Because model inaccuracy originates from a deficiency of information, researchers rely on physical principles to further refine structures. In recent CASP (Critical Assessment of techniques for protein Structure Prediction) blind prediction experiments, a model refinement category was introduced to evaluate existing model refinement methods and stimulate advances in the field (7).

The GalaxyRefine web server for protein structure refinement (8) was released on the GalaxyWEB server (9,10) in 2013. This server is based on a refinement method that performs short molecular dynamics (MD) relaxations after repeated side chain repacking perturbations. The GalaxyRefine server has been widely used in both experimental and computational studies. For instance, the server has been used by experimentalists in functional studies involving protein modelling to improve the quality of model structures obtained using other prediction methods (11–14). Additionally, developers of computational algorithms have combined the server algorithm with prediction methods employed in other research areas for improving prediction quality (15–17).

Here, we present GalaxyRefine2, an upgraded version of GalaxyRefine (8), which reflects a progress made during recent CASP experiments (18). In contrast to the previous version that focused on refinement in a local environment by a local move set, side chain repacking, GalaxyRefine2 introduces various local and global move sets and accumulates the conformational changes iteratively, enabling larger movements. The local and global move sets utilize estimated structure error to focus on refinement efforts in more inaccurate regions. Available structures of homologous proteins in the structure database are also used to enrich the possible structure pool (19). A benchmark test of CASP refinement targets showed that GalaxyRefine2 can improve model structures by 2-fold compared to GalaxyRefine in terms of the accuracy measures GDT-HA (20) and LDDT (21).

## THE GALAXYREFINE2 METHOD

### Overall protocol

**Figure 1.**
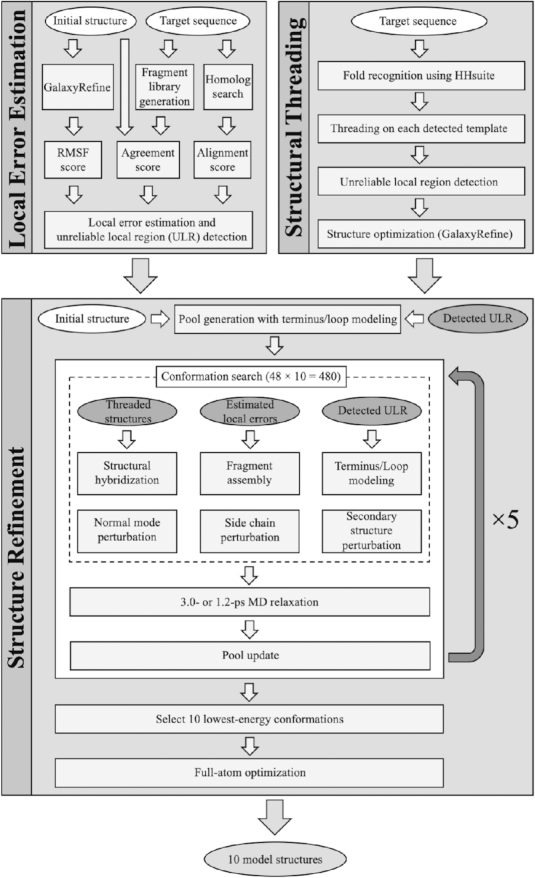
Flowchart of the GalaxyRefine2 protocol. The protocol consists of two pre-processing steps and the main refinement step.

The computational protocol of GalaxyRefine2 is schematically shown in Figure 1. The protocol is a light version of the method presented in (19), with a smaller number of iterations. Details can be found in the reference, and the method is summarized below.

### Pre-processing: local error estimation

Residue-wise error of the input structure is first estimated based on RMSF, FRAG and MSA scores. The RMSF score is residue-wise root-mean-square fluctuation in 24 runs of 14.4-ps MD relaxation involving side chain repacking every 1.2 ps (8). The FRAG score measures the agreement between backbone torsion angles of the input structure and those of fragments in the fragment library. The MSA score is the average of the position-specific scoring matrix components (22) from a multiple alignment of the sequences of homologs detected through HHsearch (23) against the input sequence. The alignment is generated by PROMALS3D (24). Residue-wise error is predicted using a linear model that combines these three scores. Finally, stretches of consecutive residues with high estimated errors are designated as unreliable local regions (ULRs).

### Pre-processing: structure threading

Among the structures of detected homologs, only those with TM-score >0.5 (25) to the input structure are considered in this step. The input sequence is threaded onto each homolog structure based on the alignment of PROMALS3D (24). The threaded structure undergoes local error estimation and structure optimization by the GalaxyRefine algorithm (8). Local patches of the optimized structure, which are not part of the predicted ULRs are used by the ‘structure hybridization’ operator in the refinement step.

### Structure refinement

An initial pool of 48 structures is generated from the input structure by re-building the termini and loops predicted as ULRs. At each iteration cycle, 480 trial structures are generated by applying structure operators 10 times to each pool structure. Structure operators include three operators that drive local refinement, ‘fragment assembly’, ‘loop modelling’ and ‘side chain perturbation’, and three that drive larger changes, ‘normal mode perturbation’, ‘structural hybridization’ and ‘secondary structure perturbation’. ‘Fragment assembly’ re-builds regions with higher estimated errors through fragment assembly and triaxial loop closure (26,27). ‘Loop modelling’ either mixes the backbone torsion angles of a selected ULR with those of another pool structure or mutates them. ‘Side chain perturbation’, as in GalaxyRefine, repacks side chains (8). ‘Normal mode perturbation’ perturbs the structure toward one of low-frequency normal modes. ‘Structure hybridization’ hybridizes the structure with the structures threaded to homologs. ‘Secondary structure perturbation’ perturbs the orientations of the secondary structure chunks in a stochastic manner.

The 480 structures are then locally optimized by a 3.0-ps MD relaxation (1.2-ps after loop modelling). Each low-energy trial structure replaces a pool structure with higher energy that is structurally close enough. If a low-energy trial structure is not close to any pool structures, the highest energy pool structure is replaced. The criterion of closeness is gradually increased with iterations to facilitate broad sampling (19). After five iteration cycles, all 2400 generated structures are scored, and the 10 lowest-energy structures are selected. The 10 structures are subject to full-atom optimization to improve their stereochemical properties and are reported as the final refined structures.

### Energy function

The energy function used for MD relaxation is a linear combination of physics-based energy, statistical potentials and restraints, as described in (19). The restraints are derived from the input structure in terms of the Cartesian coordinates of alpha carbons and pair distances between alpha carbons or between backbone nitrogen and oxygen. To reduce the tendency of being restrained too strongly to the initial structure, higher 10% restraints are neglected during relaxation (28). The user can select the functional form of the restraints from either harmonic or Lorentzian. The default option of GalaxyRefine2 is Lorentzian, which allows for wider sampling than the harmonic form. The user may select the harmonic form by choosing the ‘Conservative’ option if the input structure is known to be reliable and only local refinement is desired. Energy without restraints is used in final scoring.

### Method performance

**Figure 2.**
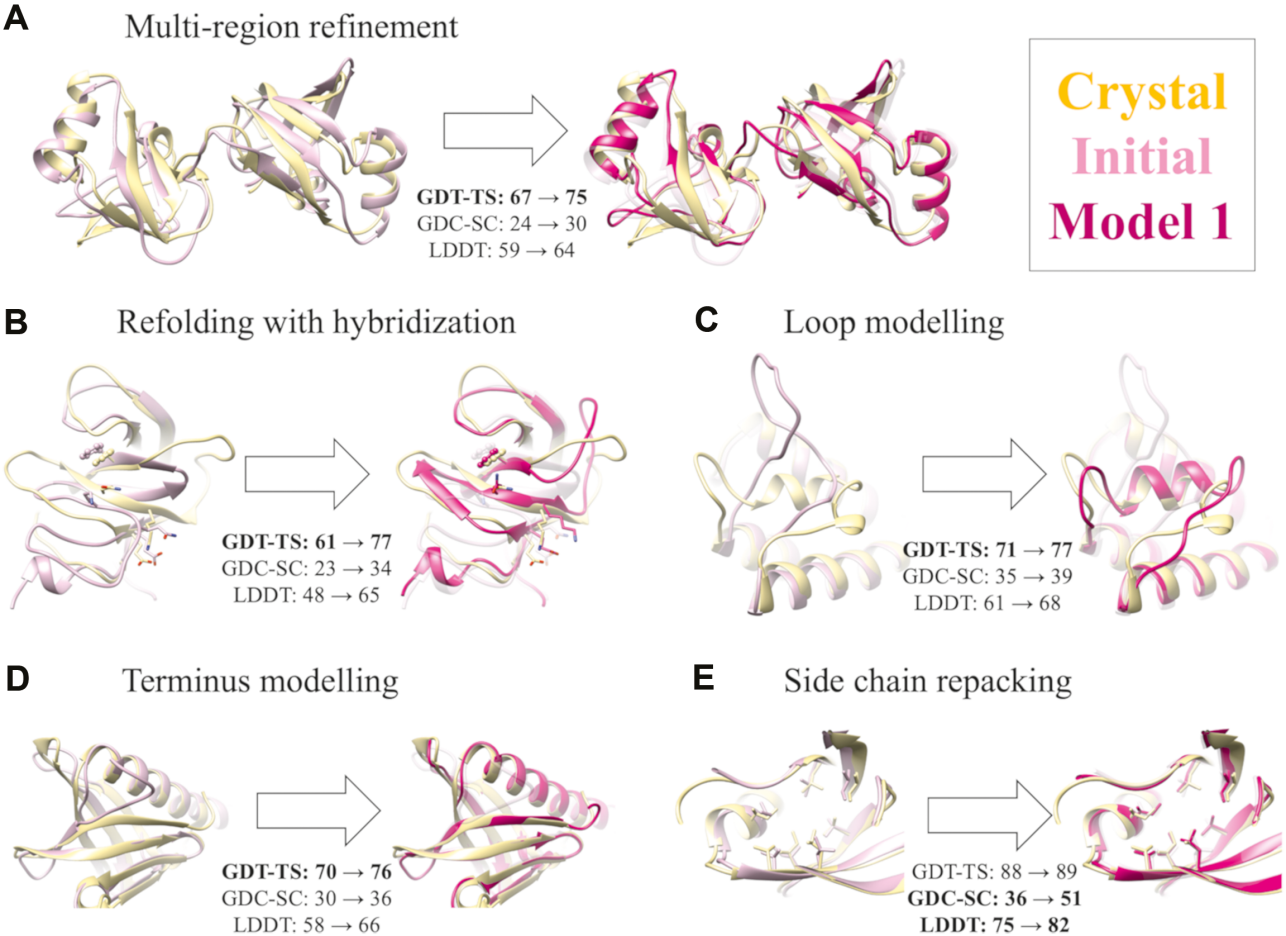
Successful refinement examples from CASP benchmark set. GalaxyRefine2 can improve structures at both the global level (**A**: TR462 and **B**: TR896) and local level (**C**: TR948, **D**: TR614 and **E**: TR488) by applying various structure operators simultaneously.

The GalaxyRefine2 server was tested in the refinement category of CASP12 and CASP13 in a blinded manner, and the server named ‘Seok-server’ was ranked highly among all servers (18,29). The CASP13 results (http://www.predictioncenter.org/casp13/) are summarized in Table 1. GalaxyRefine2 was also compared to GalaxyRefine (8) on 114 refinement targets of CASP8-12 that are monomeric and have no missing residues in the middle. Homolog structures with a sequence identity of >40% were excluded during local error estimation and structure threading. As summarized in Table 2, the new version of GalaxyRefine, GalaxyRefine2, showed improved performance. The average magnitudes of improvement were 2-fold those of GalaxyRefine in terms of GDT-HA (20) and LDDT (21), when the server was run in the default mode. Several successful refinement examples improving models in various aspects are also illustrated in Figure 2.

**Table 1.** Performance comparison of server groups participated in CASP13 refinement category

Group names	Mean improvement of Model 1 / Best among Model 1–5^a^			
	GDT-HA	GDC-SC	LDDT^b^	– MolProbity
Seok-server (GalaxyRefine2)	+1.46 / +2.68	+3.45 / +5.06	+2.55 / +3.21	+1.47 / +1.59
Bhattacharya-Server (29)	–0.37 / +1.75	+1.70 / +3.55	+0.86 / +1.79	+1.19 / +1.34
YASARA^c^	–1.21 / –1.21	+1.69 / +1.69	+0.57 / +0.57	+1.60 / +1.60
MUFold_server	–2.28 / –1.54	–0.69 / +1.17	–0.63 / –0.26	–0.40 / –0.17
3DCNN	–11.44 / –3.28	–6.52 / –1.05	–6.83 / –3.43	+0.65 / +0.87

^a^All evaluation values were obtained from CASP official homepage, http://predictioncenter.org/casp13/results.cgi.

^b^LDDT values were re-scaled to the range of [0, 100].

^c^YASARA group submitted only one model per target.

**Table 2.** GalaxyRefine2 benchmark test results for CASP8–12 refinement targets

Methods	Mean improvement of Model 1 / Best among Model 1–10		
	GDT-HA	GDC-SC	LDDT^a^
GalaxyRefine2 (default)	+0.92 / +2.72	+1.48 / +3.69	+1.58 / +2.43
GalaxyRefine2 (conservative)	+0.92 / +1.98	+0.98 / +2.11	+1.03 / +1.47
GalaxyRefine (8)	+0.58 / +1.41	+1.14 / +2.47	+0.73 / +1.16

^a^LDDT values were re-scaled to the range of [0, 100].

## GALAXYREFINE2 SERVER

### Hardware and software

The server runs on a cluster of four Linux servers of 2.20-GHz Intel Xeon E5-2650 v4 12-core processors. The web application uses the Python programming language and the MySQL database. The GalaxyRefine2 pipeline is implemented using Python. The refinement method is implemented as part of the GALAXY program package (9,10) written in Fortran 90. JavaScript Protein Viewer (http://biasmv.github.io/pv/) is used to visualize the refined models.

### Input and output

**Figure 3.**
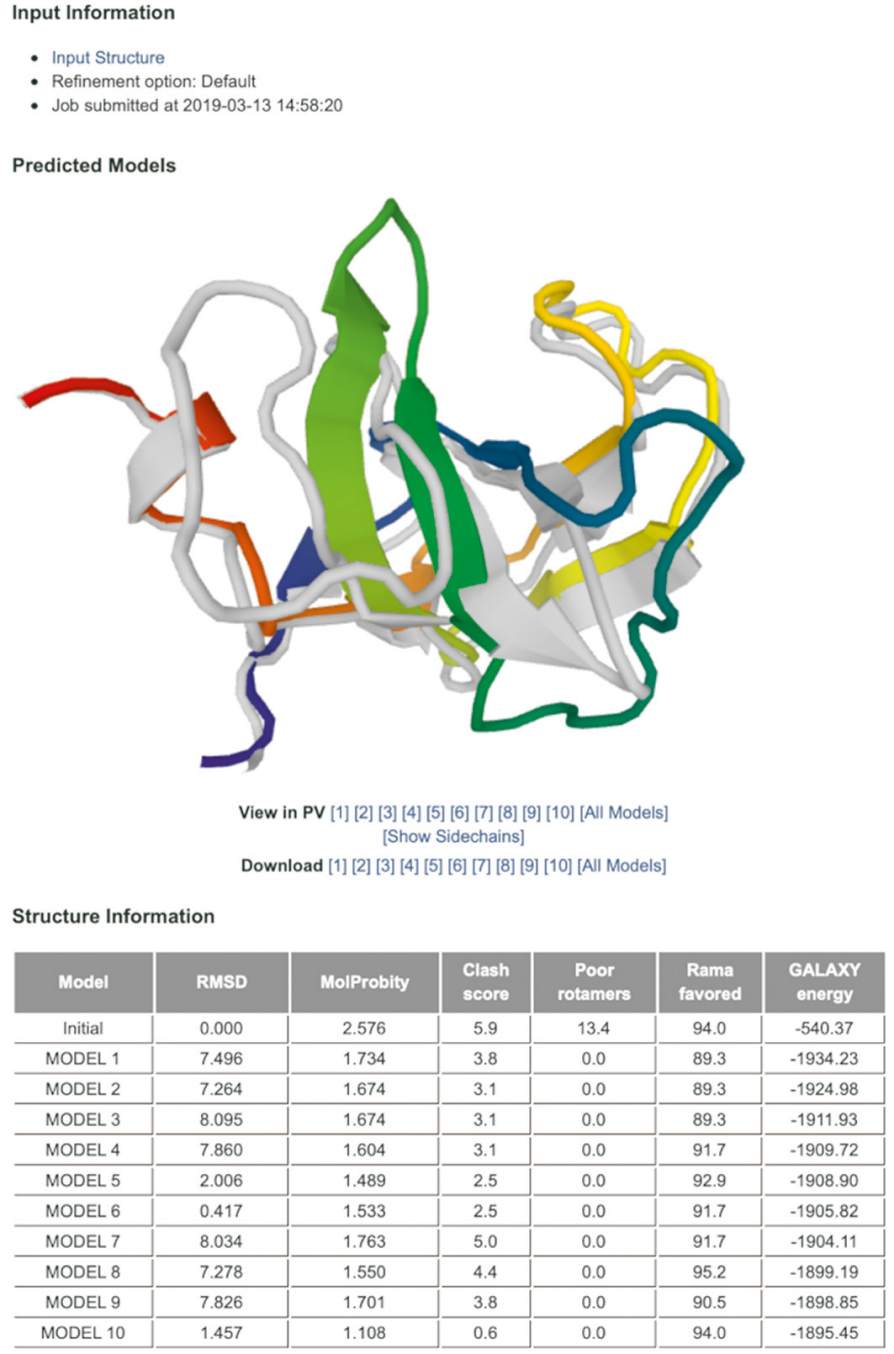
Example output page of GalaxyRefine2. Ten generated models are visualized using the JavaScript Protein Viewer. The models are downloadable in PDB format. Information such as structural changes from the input structure and MolProbity score is shown in the table.

The required input is a protein monomer structure in PDB format. The number of residues in the input file is limited to 300 for computational efficiency. Structures with missing residues in the middle are not allowed. The user may choose to run the server in the conservative refinement mode when the backbone structure of the input is considered very reliable, such as in the cases of NMR structures or template-based models generated from templates with high sequence identities. The average run time is 6–10 h. Ten refined structures, ranked based on the energy, are visualized in the web browser and are downloadable in the PDB format. Information on structural changes between the input structure and refined structures is provided in terms of RMSD and MolProbity score (Figure 3).

## CONCLUSIONS

GalaxyRefine2, an updated version of GalaxyRefine (8), refines local and global protein structures simultaneously by iterative conformational sampling, unlike GalaxyRefine, which was limited to local refinement. GalaxyRefine2 was successful in conducting blind prediction in CASP12 (18) and CASP13 refinement experiments. This server can, therefore, be used to improve predicted protein structures or low-resolution experimental structures for further interpretations or applications.

## Notes

Present address: Gyu Rie Lee, Department of Biochemistry, University of Washington, Seattle, WA 98195, USA.

Present address: Lim Heo, Department of Biochemistry and Molecular Biology, Michigan State University, East Lansing, MI 48824, USA.
